# Structural and molecular basis of mismatch correction and ribavirin excision from coronavirus RNA

**DOI:** 10.1073/pnas.1718806115

**Published:** 2017-12-26

**Authors:** François Ferron, Lorenzo Subissi, Ana Theresa Silveira De Morais, Nhung Thi Tuyet Le, Marion Sevajol, Laure Gluais, Etienne Decroly, Clemens Vonrhein, Gérard Bricogne, Bruno Canard, Isabelle Imbert

**Affiliations:** ^a^Centre National de la Recherche Scientifique, Aix-Marseille Université, CNRS UMR 7257, Architecture et Fonction des Macromolécules Biologiques, 13009 Marseille, France;; ^b^Global Phasing Ltd., Cambridge CB3 0AX, England

**Keywords:** RNA virus, virus replication, proofreading, ribavirin, fold evolution

## Abstract

Coronaviruses (CoVs) stand out among RNA viruses because of their unusually large genomes (∼30 kb) associated with low mutation rates. CoVs code for nsp14, a bifunctional enzyme carrying RNA cap guanine N7-methyltransferase (MTase) and 3′-5′ exoribonuclease (ExoN) activities. ExoN excises nucleotide mismatches at the RNA 3′-end in vitro, and its inactivation in vivo jeopardizes viral genetic stability. Here, we demonstrate for severe acute respiratory syndrome (SARS)-CoV an RNA synthesis and proofreading pathway through association of nsp14 with the low-fidelity nsp12 viral RNA polymerase. Through this pathway, the antiviral compound ribavirin 5′-monophosphate is significantly incorporated but also readily excised from RNA, which may explain its limited efficacy in vivo. The crystal structure at 3.38 Å resolution of SARS-CoV nsp14 in complex with its cofactor nsp10 adds to the uniqueness of CoVs among RNA viruses: The MTase domain presents a new fold that differs sharply from the canonical Rossmann fold.

## Significance

Emerging coronaviruses (CoVs; severe acute respiratory syndrome-CoV and Middle East respiratory syndrome-CoV) pose serious health threats globally, with no specific antiviral treatments available. These viruses are able to faithfully synthesize their large genomic RNA. We report, however, that their main RNA polymerase, nsp12, is not accurate. To achieve accuracy, CoVs have acquired nsp14, a bifunctional enzyme able to methylate the viral RNA cap [methyltransferase (MTase)] and excise erroneous mutagenic nucleotides inserted by nsp12. Strikingly, ribavirin can be excised from the viral genome, thus showing no antiviral activity. The crystal structure of nsp14 shows that it is unique, having been replaced by other MTase types during evolution. This unprecedented RNA correction machinery has allowed RNA genome size expansion, but also provided potential nucleoside drug resistance to these deadly pathogens.

Faithful transmission of genetic information is central to all living organisms. In the RNA virus world, RNA-dependent RNA polymerases (RdRps) lack co- and postreplicative fidelity-enhancing pathways, and final RNA genome copies incorporate mutations at a much higher rate than that observed for DNA genomes (1). The low fidelity accompanying viral RNA genome synthesis is mostly responsible for viral RNA genome diversity. The best-adapted genomes are subsequently selected in the host cell; thus, diversity is essential for both viral fitness and pathogenesis (2, 3). The difference in replication fidelity between DNA- and RNA-based genomes has been correlated to the difference in their genome size. RNA virus genomes range from ∼3–32 kb in size; DNA-based organisms with genome sizes up to several hundred megabases necessitate a replication machinery that is much more accurate than that of their viral counterparts (3). The low fidelity of RdRps has been exploited as an Achilles heel to combat viral infections since a promiscuous substrate choice renders the RdRps prone to incorporate nucleotide analogs into viral RNA. This family of molecules constitutes one of the cornerstones of antiviral strategies. Many RNA viruses are sensitive to ribavirin (Rbv), a mutagenic guanosine analog discovered more than 40 y ago (4). Rbv exerts its antiviral activity through numerous and possibly nonmutually exclusive mechanisms, which are not fully understood (5). Among those mechanisms, Rbv 5′-triphosphate (Rbv-TP) serves as a substrate for the viral RdRp and is incorporated into the nascent viral genome. The ambiguous coding nature of its purine-mimicking nucleobase accounts for its antiviral effect through lethal mutagenesis (6). Additionally, Rbv 5′-monophosphate is a potent inhibitor of the cellular enzyme inosine monophosphate dehydrogenase (IMPDH). IMPDH inhibition depresses cytoplasmic GTP pools, thus affecting RNA polymerase fidelity through nucleotide pool imbalance. Importantly, in contrast to hepatitis C virus (7), respiratory syncytial virus (8), and Lassa fever (9) infections, coronavirus (CoV)-infected patients do not respond to Rbv (10, 11).

CoVs include two highly pathogenic viruses responsible for severe acute respiratory syndrome (SARS) (12) and Middle East respiratory syndrome (MERS) (13), for which neither treatment nor a vaccine is available. They possess the largest genome among RNA viruses. The CoV single-stranded (+) RNA genome carries a 5′-cap structure and a 3′-poly (A) tail (14). Replication and transcription of the genome are achieved by a complex RNA replication/transcription machinery, made up of at least of 16 viral nonstructural proteins (nsps). The CoV replication/transcription complex harbors a wide variety of RNA processing activities, with some being typically found in RNA viruses, such as RdRp (nsp12) and helicase (nsp13) (15). Nsp12-RdRp requires a processivity factor made of the CoV nsp7/nsp8 complex (16). Other activities are generally found in RNA viruses with a cytoplasmic life cycle, such as methyltransferase (MTase) activities involved in RNA cap modification. An N7-MTase activity resides in the carboxyl (C) terminus domain of nsp14 (17) and a 2′O-MTase activity was identified in nsp16 (18). Interestingly, additional processing activities are found and are less common in RNA viruses, such as an endoribonuclease (nsp15) (19, 20) that is involved in innate immune response evasion (21) and a 3′-5′ exoribonuclease (ExoN) in the amino (N) terminus of nsp14 (22).

Outside CoVs and other members of the Nidovirales order, 3′-5′ ExoN activity is only found in the Arenaviridae family (23). For example, the Lassa virus carries 3′-5′ ExoN activity involved in immune suppression (24). Among nidoviruses, 3′-5′ ExoN activity is found in all large-genome nidoviruses (in addition to CoVs, toroviruses, and roniviruses, with 28-kb and 26-kb genomes, respectively), as well as in mesoniviruses (∼20 kb). Short-genome nidoviruses (arteriviruses, ∼13–15 kb) lack such ExoN activity (25). Acquisition of such ExoN activity might have allowed nidoviruses to evolve larger genomes (26). Moreover, since nsp14-ExoN belongs to the DEDD 3′-5′ exonuclease superfamily (22), which includes DNA proofreading enzymes, this activity was proposed to be involved in a CoV RNA proofreading mechanism (15). Indeed, genetic inactivation of nsp14-ExoN of the CoV murine hepatitis virus and the SARS-CoV results in 15-fold and 21-fold more mutations in genomes than for wild-type (wt) viruses in infected cells, respectively (27, 28). Consistent with these results, a wt nsp14-ExoN proofreading activity protects SARS-CoV from the deleterious effect of 5-fluorouracil (5-FU) (29). Hence, inactivation of the ExoN activity sensitizes the virus to 5-FU, leading to a 14-fold increase in genomic mutations relative to wt (29).

Nsp14 can bind to several viral partners. It can associate with the polymerase complex (nsp12/nsp8/nsp7), and the resulting complex retains all associated enzymatic activities (i.e., RdRp, ExoN, N7-MTase) (16). It can also associate with another CoV protein, nsp10, and the resulting complex shows in vitro a strongly enhanced ExoN activity (30). The nsp10/nsp14 complex strictly targets double-stranded (ds) RNA, excising nucleotides in the 3′-5′ direction. Interestingly, this complex can also selectively remove a mismatched nucleotide mimicking a misincorporated ribonucleotide at the 3′-end of a dsRNA substrate (30). A crystal structure of the SARS-CoV heterodimer nsp10/nsp14 was recently determined (31). Nsp14 is organized in two functional domains: the ExoN domain at its N terminus, which interacts with nsp10, and the N7-MTase domain at its C terminus. The crystal structure redefined the ExoN catalytic residues as being of the DEED type, and unveiled two previously unknown zinc finger motifs in the N7-MTase domain.

In the present study, we have solved the SARS-CoV nsp14 crystal structure. We show that the molecule flexibility is essential in the interaction with the viral polymerase. We visualize conformational changes responsible for nsp10-mediated ExoN stimulation using X-ray crystallography and small-angle X-ray scattering (SAXS) methods. We have reconstituted the CoV RNA proofreading pathway, by which a ribonucleotide misincorporated into RNA is excised by an active replicase complex. Rbv 5′-monophosphate is incorporated at the 3′-end of RNA by the SARS-CoV polymerase. It is also efficiently excised by the nsp14-ExoN activity, which acts as a proofreading activity. Because of its dual role in synthesis and repair of viral RNA, this multifunctional replicase complex represents an attractive target for designing novel compounds active against CoVs. Finally, the crystal structure reveals that the nsp14 N7-MTase domain, strikingly, does not belong to the canonical Rossmann fold MTase family, making nsp14 a unique RNA processing enzyme and giving it a central role in large RNA genome evolution.

## Results

### Structural Overview of SARS-CoV nsp10/nsp14 Complex.

**Fig. 1.**
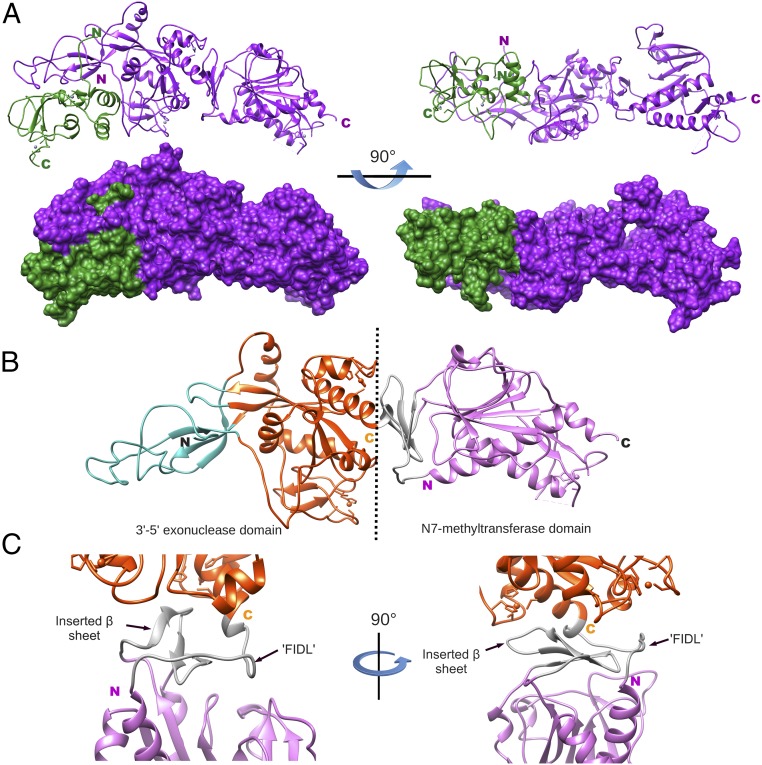
Structure model of SARS-CoV nsp10/nsp14 complex. (*A*, *Top*) Ribbon structures of nsp10 (green) and nsp14 (purple) from a side view and 90° rotation with respect to the side view. Amino terminus and carboxyl terminus extremities of each protein are indicated by the letters N and C with their respective colors. (*A*, *Bottom*) Surface of the nsp10/nsp14 complex structure in similar orientation as the top view. (*B*) Ribbon structure of nsp14 shown as colored subdomains: cyan (nsp10-binding site), orange (exonuclease), gray (flexible linker or hinge), and purple (N7-MTase). Amino and carboxy domains are separated by a black dashed line, and terminal extremities are indicated by the letters N and C (black). (*C*) Closeup view of the nsp14 hinge region: carboxyl extremity of the exonuclease (orange), hinge (gray), and amino extremity of the N7-MTase (purple). The hinge region is formed by the flexible interdomain loop (‘FIDL’) wrapping around the β-sheet protruding from the MTase domain (inserted β-sheet). Right and left orientations are rotated 90°.

We determined the crystal structure of the nsp10/nsp14 heterodimer by multiwavelength anomalous diffraction (MAD) using the anomalous scattering signal of zinc (Fig. 1*A*). The crystal belongs to the space group P2_1_2_1_2_1_, with cell dimensions a = 185.97 Å, b = 189.74 Å, c = 195.93 Å, and α = β = γ = 90°, and containing four heterodimers in the asymmetrical unit (Table S1). The four heterodimers assemble in a tetrahedron-like structure (Fig. S1*A*), while the higher order of the crystal shows that the structural assembly forms a loose mesh crossed by fairly large elliptical solvent channels for which the dimension of the major axis is ∼120 Å and that of the minor axis is ∼50 Å (Fig. S1*B*).

Nsp14 is a modular protein composed of two functional domains, bordered on their N terminus by two other structural domains. These four regions are organized as follows (Fig. 1*B*): (*i*) a flexible N terminus forming the major part of contacts with nsp10 (cyan), followed by (*ii*) the exonuclease domain (orange), (*iii*) a flexible hinge region formed by a loop and three strands (gray) (Fig. 1*C*), and (*iv*) a C terminus N7-MTase domain (pink). The surface defined by the C terminus end of the ExoN domain (i.e., hinge, N7-MTase domain) is reminiscent of a saddle, which may accommodate a protein partner (discussed below). The nsp14 structure compared with the one published (31) presents insightful modifications. Despite identical topology compared with the other available structure, nsp14 presents a high level of flexibility. Hence, overall nsp14 chain-to-chain superimposition presents significant root-mean-square deviations (rmsds) drifting from 1.13 to 4.10 Å. Moreover, we also observe significant mobility between the two domains of nsp14 (discussed in *The Hinge Region Allows Large Movements Between the ExoN and N7-MTase Domains*), while the structure of nsp10 remains essentially unchanged with rmsds ranging from 0.68 to 0.95 Å, relative to published structures of nsp10 alone (32, 33) or in complex with nsp14 (31) or nsp16 (34, 35).

### Structure of the nsp14-Exonuclease Domain.

**Fig. 2.**
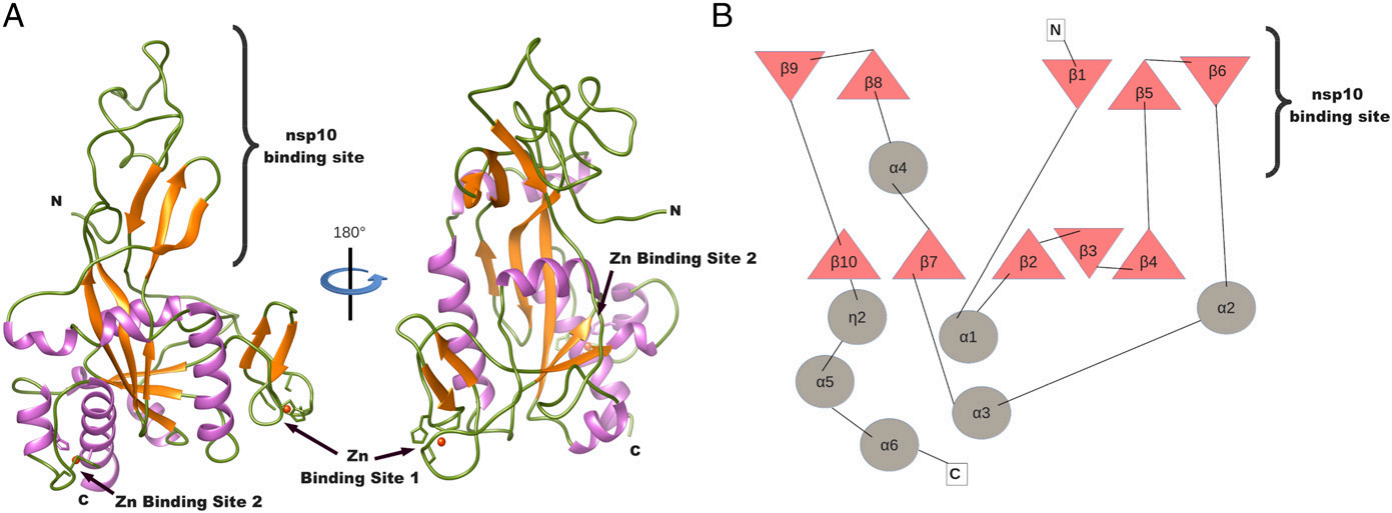
Structure model and topology of nsp14 exonuclease domain. (*A*) Ribbon model of the exonuclease domain colored by secondary structures: loop (green), α-helix (purple), and β-strand (orange). The two zinc-binding sites are shown with a zinc atom (red sphere). (*B*) Topology of nsp14-ExoN domain: β-strand (triangles) and α-helix (circles). The area of interaction of the nsp14-ExoN domain with nsp10 is indicated.

The ExoN domain is an α/β fold constituted of six α-helices and 10 strands, organized into three distinct β-sheets (Fig. 2*A*). The core of the domain is formed by a central β-sheet made up of five antiparallel strands (β10, β7, β2, β3, and β4) and surrounded by five helices (α1, α2, α3, α4, and α6). Despite a pronounced twist of the central sheet, the fold is reminiscent of that of the DEDD exonuclease superfamily (36) (Fig. 2*B*). A Protein Data Bank fold search on the SARS-CoV ExoN domain retrieves the structures of the human 3′-5′ ExoN (37) and the *Pseudomonas aeruginosa* ribonuclease T (38), both with an rmsd of 1.2 Å. From this central domain, between the β4 and α2 spike, a β-hairpin structure containing β5 and β6 (residues 122–138) forms with β1 a second antiparallel β-sheet–binding nsp10. The third antiparallel β-sheet grows out of the base of the central core domain between α4 and β10. It is made up of β9 and β8, and presents at its base a first zinc finger motif comprising residues Cys207, Cys210, Cys226, and His229. A second zinc finger located between α5 and α6 is formed by residues His257, Cys261, His264, and Cys279.

### Nsp10 Interacts with nsp14, Thereby Stabilizing the ExoN Active Site.

The entire nsp10 peptide chain was built, unlike previously reported structures of nsp10 solved either alone (32, 33) or in complex with nsp16 (34, 35). Here, the N terminus of nsp10 is stabilized by the first 25 residues of nsp14, forming a clamp that accommodates the first 10 residues of nsp10. The interaction between nsp10 and nsp14 is figuratively similar to a hand (nsp14) over fist (nsp10). Fingers are formed by the whole flexible N terminus region of nsp14 (residues 1–50), β1 (residues 51–55), and an antiparallel β-strand budding out from the ExoN domain (residues 122–138), while the palm is constituted by top and side (residues 55–69 and 195–202) of the ExoN domain (Fig. 1). Consequently, in this analogy, the cavity holding the active site of the exonuclease domain is located on the back of the palm and the interaction surface of nsp10 with nsp14 is substantially wider than that observed for nsp10/nsp16 (34, 35). Indeed, the nsp10/nsp14 interaction surface is of 7,798 Å^2^ versus 3,225 Å^2^ for nsp10/nsp16.

This particular mode of interaction of nsp10 with nsp14 suggests how nsp14-ExoN activity is stimulated by nsp10 (30). In the presence of nsp10, residues of the ExoN active site are correctly positioned and form a highly active ExoN, as judged by the observed distance between catalytic residues. In the absence of nsp10, using SAXS (*SI Text* and Fig. S2), we observed large conformational changes in the N terminus of nsp14, which impact the overall shape of the exonuclease fold (Fig. S3). Without the fist (nsp10), the fingers of the hand fold onto the palm; by doing so, they distort the cavity containing the ExoN catalytic residues. This cavity is located in the back of the palm, by moving up the α-helix (residues 147–159) forming the top of the RNA-binding path, with distant residues (residues 110–117) repositioning the β-sheet. This distortion indicates that the ExoN catalytic pocket partially collapses in the absence of nsp10, accounting for the weak ExoN activity of nsp14 alone (30).

### The Hinge Region Allows Large Movements Between the ExoN and N7-MTase Domains.

**Fig. 3.**
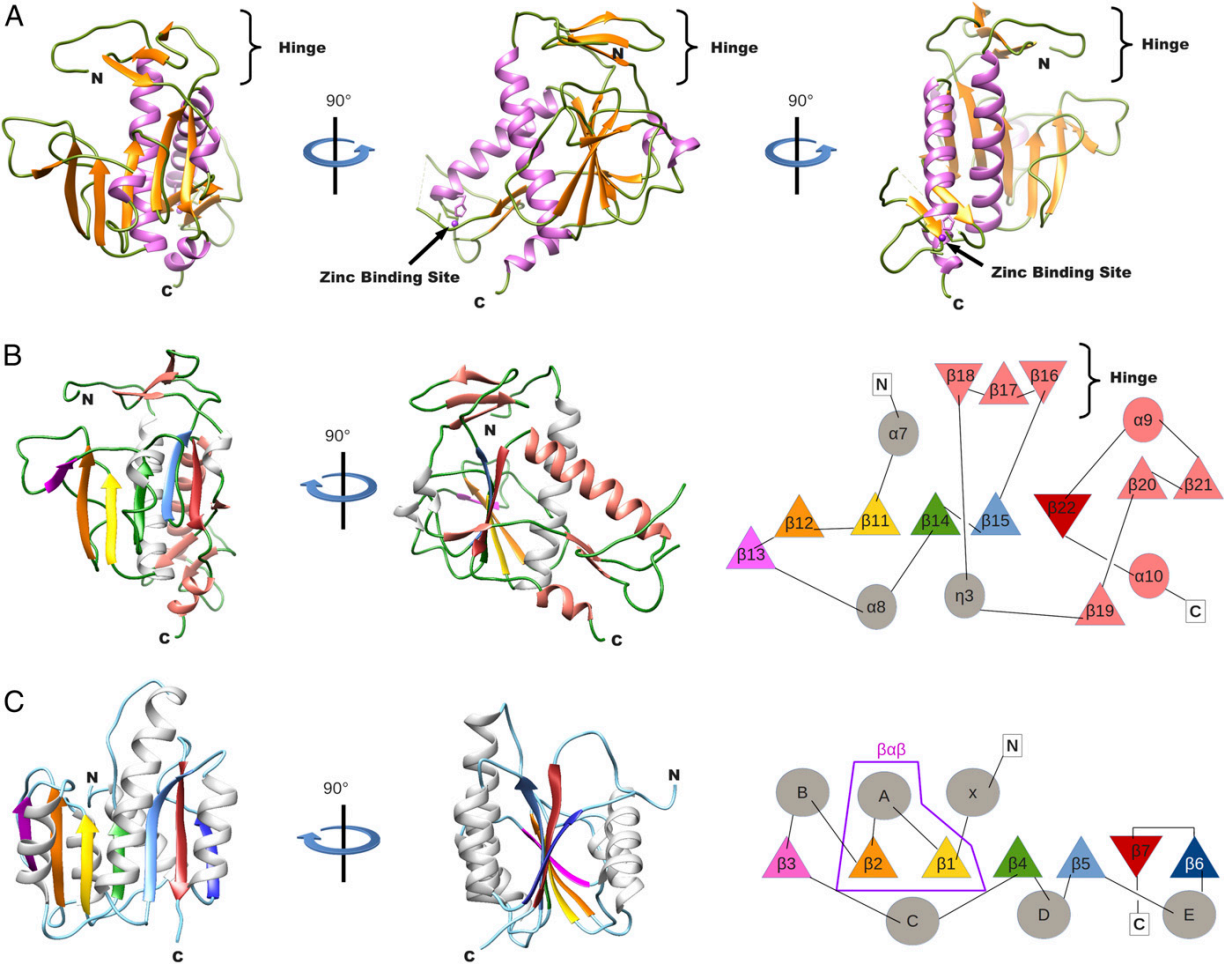
Structure model and topology of the nsp14 N7-MTase domain. (*A*) Ribbon model of the N7-MTase domain in three orientations (90° rotation apart) and colored by secondary structures as in Fig. 2*A*. The third zinc-binding site is shown with a zinc atom (purple sphere). (*B*) Organization analysis of the nsp14 N7-MTase domain. (*Left*) Ribbon model of the MTase domain in two orientations (of 90° rotation). Secondary structures are colored to highlight the topology: loop (green), α-helix (gray and salmon), and β-strand (purple, orange, gold, green, light blue, red, and salmon). (*Right*) Topology diagram of the nsp14 N7-MTase domain: β-strand (triangles) and α-helix (circles), with the corresponding secondary structures following the same color code as for the ribbon presentation. (*C*) Organization analysis of a canonical Rossmann fold FtsJ MTase (60). (*Left*) Ribbon model in two orientations (of 90° rotation), with the same color code as in *B*. (*Right*) Standard topology of MTase (so-called Rossmann fold). Secondary structures that align with those in the nsp14 N7-MTase domain have the same color as used in the *B*. The structural motif β-α-β defining the Rossmann fold is surrounded in purple.

The ExoN and N7-MTase domains of nsp14 are separated by a hinge region that allows significant movements between both domains (Fig. 1 and Fig. S3). The hinge, which can be seen as an extension of the N7-MTase domain, is composed of a flexible interdomain loop (residues F286–G300) (Fig. 1*C*) wrapping around a three-stranded antiparallel β-sheet (β18, β17, and β16, including residues L406–A430) jutting out of the N7-MTase domain (Fig. 3 *A* and *B*). Sequence analyses show that amino acids forming the flexible hinge are conserved across Coronavirinae, suggesting that they play a critical and functional role in regulation of the nsp14 hinge region movement. Indeed, this particular structure allows lateral and rotational movements of the C-terminal domain with respect to the N-terminal domain, such that an ∼13 Å deviation is observed from one peptide chain to the other (crystallography and SAXS analysis in Fig. S3).

### The nsp14 N7-MTase Domain Is Not a Rossmann Fold.

The nsp14 N7-MTase domain comprises a total of 12 β-strands and five α-helices (Fig. 3 *A* and *B*). The fold presents a central five-stranded β-sheet made up of four parallel strands (β12, β11, β14, and β15) and one antiparallel strand (β22). The central β-sheet is sandwiched between a single α-helix (α7) and three long loops punctuated by two small helices (α8 and η3). The central sheet is surrounded by two strands (β13 and β19), which are perpendicular but not fully aligned with the central sheet. Between β15 and the second turn, η3 is inserted into the β-sheet of the hinge (discussed above), which forms a lid above the active site. Following the turn is a long flexible extension drifting almost away from the β-sheet, which ends with a β-strand (β20) involved in a parallel β-sheet with another strand (β21). A long, disordered region (residues 453–465) not built in our structural model separates the two strands, and we postulate that it may serve as a binding platform for other partners (39). The strand is followed by a long helix (α9). Between the tip of strand β21 and the bottom of helix α9, a third zinc-binding site is found. It is formed by residues Cys452, Cys477, Cys484, and His487. This zinc finger constrains the structure locally and orients α9 toward the center of the structure, making α9 and α7 cross each other and positioning the C terminus of the helix just above the β-sheet, allowing strand β22 to complete the central β-sheet. The C terminus of nsp14 ends with a helix (α10) which is at almost 90° from strand β22 and, together with α9, sandwiches the parallel β-sheet (β20–β21). The molecular mechanism of the RNA cap guanine N7-methylation has been described previously (31, 40).

So far, all reported RNA cap-MTase structures belong to the Rossmann fold family (41). The Rossmann fold, defined as a βαβ structure, is one of the five most common structural motifs widely distributed and considered as remnants of most ancient protein architectures (42) (Fig. 3*C*). Briefly, the Rossmann fold can be described as a seven-stranded β-sheet with at least three parallel α-helices on each side (α/β fold). Some variations of the fold have been observed among structures of dinucleotide-binding domains: Variations include the number of β-strands in the complete β-sheet that forms the Rossmann fold, as well as the length and number of secondary structures of the segment that connects the second and third β-strands (43). Two landmarks of the Rossmann fold family are the first structural feature, βαβ, and the motif sequence G-x-G-(x)_n_-G, which both constitute the NTP-binding site. It is the conservation of the canonical βαβ fold motif in these enzymes that ascertains a common evolutionary origin of these proteins.

The SARS-CoV nsp14 N7-MTase domain is thus an exception, as the structure reveals major structural deviations incompatible with classification into this family (compare Fig. 3 *B*, *Right* and *C*, *Right*). The most conserved feature of the Rossmann fold family, the βαβ structural motif, is absent, as are standard MTase motifs in the protein sequence, explaining why it had escaped detection upon sequence alignment-based investigations (15). The SARS-CoV N7-MTase domain exhibits a unique fold, defining an MTase representative that should nucleate a new structural family.

### The nsp12-RdRp Interacts with both the ExoN and N7-MTase Domains of nsp14.

Nsp10 interacts with nsp14 which, in turn, interacts with nsp12-RdRp, albeit without direct interaction of nsp10 with nsp12-RdRp (44). The interaction surface between nsp10 and nsp14 has been mapped using mutagenesis (45) and structural studies (this study and ref. 31). However, the structural basis of the interaction of nsp14 with nsp12-RdRp is still unresolved. Based on our nsp14 structure, we defined three nsp14 domains and showed that not only the ExoN domain but also the N7-MTase domain of nsp14 interacts with nsp12-RdRp and that the first 71 amino acids of nsp14 are not essential for interaction with the polymerase (Fig. S4).

Then, we probed the interaction of the nsp14 saddle-shaped surface (Fig. 1*A*) with nsp12-RdRp. Based on both the amino acids exposed to the surface of the saddle and sequence conservation across Coronavirinae, 14 mutants in the nsp14 N7-MTase domain were generated (Y296A, P297A, N306A, R310A, V466K, L468K, K469A, C473A, T475A, L479A, H487A, L495A, Y498A, and N499A). As a control, the nsp14-ExoN catalytic site mutant (D90A/E92A) was also included in the study. The propensity of these nsp14 mutants to interact with nsp12-RdRp and their respective ExoN and N7-MTase activities were determined as previously described (18, 30, 44). As shown in Table S2, three nsp14 substitutions (R310A, H487A, and L495A) significantly alter N7-MTase activity (1% ± 0.1, 14% ± 2.5, and 26% ± 0.7, respectively). The critical role of the R310 residue in cap methylation has already been demonstrated as an *S*-adenosylmethionine (SAM)-binding residue (40). Interestingly, seven residues in the nsp14 N7-MTase domain (Y296, P297, C473, T475, H487, L495, and Y498) are essential for ExoN activity (Fig. S5). These mutants are correctly folded since they retain either interaction with nsp12-RdRp (Fig. S6) or N7-MTase activity (Table S2) (except nsp14 H487A, which is altered for all three properties tested, as discussed below). Finally, two nsp14 residues (H487 and Y498) are critical for the interaction with nsp12-RdRp in vitro (Fig. S6). His487 is located in zinc finger 3 of the N7-MTase domain (Fig. S5). Whereas the nsp14 Y498A mutant conserves an N7-MTase activity in the same range as nsp14 wt (95% ± 6), the nsp14 H487A mutant exhibits a substantially reduced N7-MTase activity (14% ± 2.5). The nsp14 H487 residue may be involved in charge-mediated protein/protein interactions since the nsp14 H487R mutant fully conserved N7-MTase activity (31), consistent with the nsp14/nsp12 interaction site proposed here.

### The SARS-CoV Polymerase Complex Exhibits Low Nucleotide Insertion Fidelity.

**Fig. 4.**
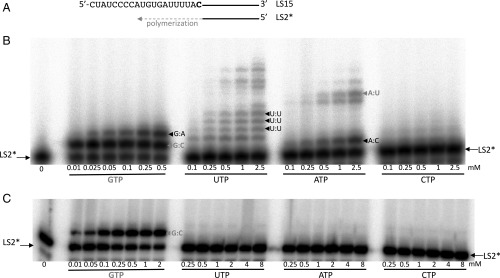
Single-nucleotide incorporation catalyzed by either the SARS-CoV polymerase complex or the DENV RdRp. (*A*) Sequence of the RNA primer/template used is shown: The 20-nt primer LS2 was 5′-radiolabeled (marked by an asterisk) and annealed to the 40-nt template LS15. The RNA template corresponds to the last 40 nt of the SARS-CoV 3′-nontranslated region before the polyA tail. (*B*) Single-nucleotide primer extension with the SARS-CoV polymerase complex (0.5 μM), LS2*/LS15 (0.5 μM), and increasing concentrations of the indicated NTP, added after 30 min of preincubation at 30 °C. Next, reactions were incubated for 18 min at 30 °C. (*C*) Single-nucleotide primer extension with DENV NS5 (1 μM), LS2*/LS15 (1 μM), and increasing concentrations of the indicated NTP. Reactions were incubated for 15 min at 37 °C. Conditions of enzyme purification and polymerase assay are described by Potisopon et al. (61). Watson–Crick base pairs are indicated in gray, and mismatches are indicated in black.

We compared the SARS-CoV polymerase complex (formed of nsp12/nsp8/nsp7) and Dengue virus (DENV) NS5 for their efficiency of single “correct” or “incorrect” nucleotide incorporation into RNA (Fig. 4). Both polymerases incorporate GMP (corresponding to the “correct” Watson–Crick base pair) efficiently into this RNA. The two polymerases, however, exhibit strikingly different fidelity. DENV NS5 does not significantly misincorporate nucleotides, even with up to 8 mM UTP, ATP, or CTP (Fig. 4*C*). This observation is in line with DENV RdRp fidelity, estimated between 1/34,000 (i.e., error rate of 3 × 10^−5^ for the U:C mispair) and 1/135,000 (i.e., error rate of 7.4 × 10^−6^ for the C:C mispair) (46). In contrast, G:A, U:U, and A:C mismatches are readily detected in the presence of the SARS-CoV polymerase complex (Fig. 4*B*). Using a variety of RNA templates, single-nucleotide primer extension opposite AMP, UMP, and GMP was similarly monitored (Fig. S7). U:G, C:A, G:U, and G:G mismatches are also readily detected (Fig. S7).

In conclusion, the SARS-CoV polymerase exhibits a nucleotide insertion fidelity in vitro that is surprisingly lower than that of DENV RdRp. It shows a general tendency to be error-prone, despite the low mutation rate observed at the virus level (28).

### The ExoN Activity Restores SARS-CoV Polymerase Nucleotide Insertion Fidelity in Vitro.

**Fig. 5.**
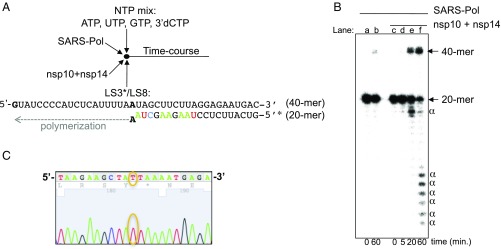
In vitro RNA proofreading by the SARS-CoV polymerase complex (SARS-Pol) and exonuclease enzymatic activities. (*A*) Schematic view of the reaction setup: SARS-Pol (0.5 μM) was incubated with the RNA primer/template LS3*/LS8 (0.5 μM) in the presence of ATP, UTP, GTP, and 3′-dCTP (500 μM each) and, once indicated, with nsp14 (100 nM) and nsp10 (300 nM). Indeed, to prevent dsRNA product degradation by the highly potent nsp10/nsp14-ExoN activity (30), the RNA template bears only a single G at its 5′-end. In the polymerase reaction, only ATP, UTP, GTP and 3′-dCTP are provided as substrates for RNA synthesis. Hence, when the radiolabeled LS3 primer is extended by the polymerase complex, only 3′-dCMP can be incorporated at the 3′-end of the extended primer, preventing nsp10/nsp14-ExoN–mediated degradation of newly synthesized dsRNA (30). Part of the primer is shown with the same color code as the sequencing of the extended primer presented in *C*. (*B*) Radiolabeled primer extension by SARS-Pol alone (lane b) or with nsp10 + nsp14 (lanes c–f). RNA products were separated by denaturing gel electrophoresis and analyzed by autoradiography. The positions of the primer (20-mer) and the full-length extension product (40-mer) are indicated. α, dsRNA product degradations by nsp14-ExoN activity. These degradation products may not have biological significance, since replication is uncoupled from excision in the present reconstituted pathway. (*C*) Part of the Sanger sequencing chromatogram of the extended LS3 from the RT-PCR products formed after incubation of LS3/LS8 with the polymerase complex, nsp10, and nsp14. The surrounding T base corresponds to the corrected base (A to U).

We analyzed the direct contribution of the SARS-CoV nsp14-ExoN activity to the proofreading of RNA synthesized by the viral polymerase. For this purpose, we used a primer/template bearing an A:A mismatch (Fig. 5*A*), which is both poorly generated (Fig. S7) and poorly extended by the SARS-CoV polymerase complex (Fig. 5*B*, lane b). We asked if the RNA polymerase may be able to resume polymerization starting from this primer/template, after proofreading by the 3′-5′ ExoN. After addition of nsp10 and nsp14, an extension product is readily detected (Fig. 5*B*, lanes e and f). We sequenced the resulting RNA products after conversion to cDNA by RT-PCR, using a specific and selective RT primer (*Materials and Methods*). With the polymerase complex alone, 100% of the clones (eight of eight) carry the initial A base. The presence of nsp10 and nsp14-ExoN promotes A-to-U repair of ∼90% of sequenced clones (43 of 49 clones) (Fig. 5*C*), whereas that of nsp10 and nsp14-ExoN(−) leaves ∼94% of sequenced clones (33 of 35 clones) unrepaired (*P* < 0.001). In line with reverse genetics findings (28), these results provide a molecular basis for RNA synthesis proofreading by the SARS-CoV replicase.

### Rbv 5′-Monophosphate Is Incorporated and Excised into/from RNA.

**Fig. 6.**
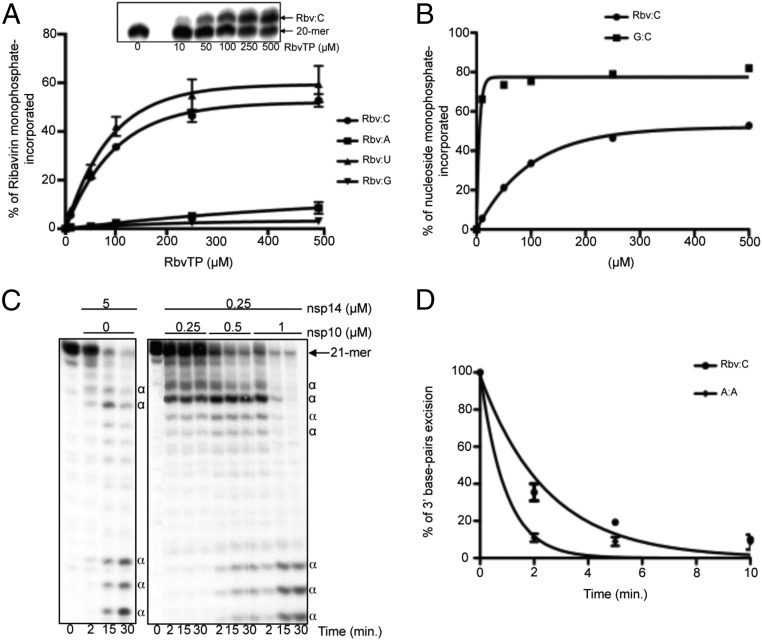
Incorporation and excision of Rbv 5′-monophosphate into/from RNA. (*A*) Incorporation efficiency of different concentrations of Rbv monophosphate (Rbv), by the SARS-CoV polymerase complex (SARS-Pol), opposite CMP, AMP, UMP, and GMP in the template, using LS2/LS15, LS2/LS1, LS2/LS17, and LS2/LS16 as the primer/template, respectively (Table S3). Single-Rbv primer extension products (21 nt) were quantified, and data were fitted to a single exponential equation. (*Inset*) Rbv incorporation by the SARS-Pol opposite CMP in the template, after denaturing gel electrophoresis and autoradiography. (*B*) Quantification of Rbv and GMP incorporation opposite CMP in the RNA template, from reactions presented in *A* and Fig. 4*B*, respectively. Data were found to fit best to the classic Michaelis–Menten equation: *V*_i_ = *V*_max_[NTP]/(*K*_m_ + [NTP]) where *V*_max_ and *K*_m_ are the maximal velocity and the affinity constant of NTP incorporation by the SARS-Pol, respectively. Using GraphPad Prism software, the Rbv-TP *K*_m_ value is 136 μM, while the GTP *K*_m_ value is 1.5 μM. (*C*) Excision of 3′-Rbv (corresponding to synthetic RNA named LS2-Rbv annealed to the RNA template LS15; Table S3) by the nsp10/nsp14-ExoN activity. The primer/template LS2-Rbv*/LS15 (0.5 μM) was incubated with either nsp14 alone (*Left*) or with increasing concentrations of nsp10 (*Right*), at 37 °C for a time course. Protein concentrations used are indicated in the panel. RNA products were analyzed as in *A*. α, dsRNA degradation products by the nsp14-ExoN activity. (*D*) Time course of excision of either Rbv:C (LS2-Rbv*/LS15 template used) or A:A (LS3*/LS8 template used) base pairs of RNA substrate in presence of nsp10 (0.5 μM) and nsp14 (0.25 μµM). The tests were done in triplicate, and an example of the data is shown in Fig. S8.

The presence of ExoN associated with low-fidelity RdRp might impact future design of anti-CoV nucleoside analogs. Is the nsp12-RdRp permissive to nucleotide analog incorporation into RNA? If yes, could the analog be excised by the associated ExoN activity? We first examined if Rbv-TP could serve as a nucleotide substrate of the SARS-CoV polymerase complex (Fig. 6*A*). Rbv 5′-monophosphate (Rbv) is incorporated into RNA, preferentially opposite template pyrimidines (UMP or CMP) (Fig. 6*A*), around 100-fold less efficiently than a regular Watson–Crick base pair (Fig. 6*B*). To determine if ExoN activity could excise Rbv from the 3′-end of a primer, we used a synthetic RNA primer exhibiting an Rbv monophosphate at its 3′ end (named LS2-Rbv) annealed to the RNA template LS15, resulting in Rbv opposite CMP (Table S3). This RNA substrate was first tested with a high concentration of nsp14 alone: A rapid disappearance of the Rbv-terminated primer is observed over time (Fig. 6*C*), confirming that an intact ribose, rather than the base, is a strong determinant of nsp14 substrate selectivity (30). The addition of increasing concentrations of nsp10 stimulates the excision rate of the terminal Rbv (Fig. 6*C*). The rate of nsp10/nsp14-mediated Rbv excision is about fourfold faster than that of an A:A mismatch (Fig. 6*D*), which are data that can be extended to any erroneous nucleotide incorporation. Indeed, it was shown that single mismatches of any type are removed by the SARS-CoV nsp10/nsp14-ExoN activity with a similar efficacy (30).

We conclude that incorporated Rbv can be readily excised by nsp10/nsp14-ExoN and, to a lesser extent, by nsp14-ExoN alone. This observation may account, at least in part, for the poor efficacy of Rbv against SARS-CoV in infected patients (10).

## Discussion

RNA polymerase fidelity (i.e., the ability to select and incorporate the correct nucleotide into an RNA strand in a template-dependent manner) is a primary determinant of mutation rates for RNA viruses. Overall, a figure of 10^−6^ to 10^−4^ substitutions per nucleotide per cell infection has been reported for viral RdRps (3). Drake (1) and Drake et al. (47) reported that mutation rates can vary by several orders of magnitude in RNA- and DNA-based organisms but that the overall mutation rate per genome stays remarkably constant, around approximately one mutation per replicated genome. Well before the discovery of nsp14-ExoN in the large Nidovirales genomes (22), this observation led to Drake’s visionary proposition that “RNA viruses would have to acquire several host genes and adapt them to RNA substrates to achieve a major reduction in spontaneous mutation rate. The result would be a substantial increase in genome size” (1).

The discovery of the involvement of CoV nsp14-ExoN in genome stability maintenance (27, 28) and the subsequent demonstrations that it binds to the nsp12-RdRp (16) and excises nucleotide mismatches (30) represent a remarkable validation of Drake’s prediction. We demonstrate here, at the molecular level, that CoVs have indeed acquired an enzyme able to enhance the overall fidelity, and that this event might have directly promoted the jump in size of CoV genomes. The acquisition and evolution of the nsp14 gene present striking features, providing structural and functional evidence that it is at a tipping point in the evolution of genetic stability during viral replication.

The N7-MTase domain of nsp14 most likely results from a de novo emergence and not from a gene duplication. The SARS-CoV nsp10/nsp14 structure was previously described as reminiscent (31) or deviant (48) of the Rossmann fold. However, based on our analysis, SARS-CoV nsp14 does not present the signature of a Rossmann fold: It lacks the defining motif βαβ (Fig. 3) and presents the structural constraint of a zinc-binding site stacking a long helix (α9) structuring the β-sheet. Moreover, a sequence analysis of SARS-CoV N7-MTase with a distant homology search only retrieves CoV sequences. Altogether, these findings indicate that the nsp14 N7-MTase domain has appeared during evolution independent from Rossmann fold MTases. It would seem logical that the nsp14 N7-MTases had appeared before Rossmann fold MTases, because the latter had successfully evolved to N7-methylate mRNA caps of all species that use the RNA cap to initiate their mRNA translations.

The nsp14 exonuclease domain has also undergone structural evolution of its fold to reversibly accommodate its cofactor nsp10. The latter has no known structural counterpart except other CoVs (32). This structural relationship between nsp10 and the exonuclease domain of nsp14 is the key to ExoN activity stimulation. When binding to the amino terminus of nsp14, nsp10 allows the exonuclease active site to adopt a stably closed conformation, allowing efficient hydrolysis of dsRNA. Conversely, in the absence of nsp10, large conformational changes in the N terminus of nsp14 occur, as shown by SAXS, leading to a weak ExoN activity (30). The potent nsp10/nsp14-ExoN activity may be involved in the degradation of immune stimulatory dsRNA, as was shown in vivo for the nsp14 of the transmissible gastroenteritis virus, a prototypic Alphacoronavirus (49). In contrast, the much lower ExoN activity of nsp14 on its own may be associated with a proofreading mechanism.

**Fig. 7.**
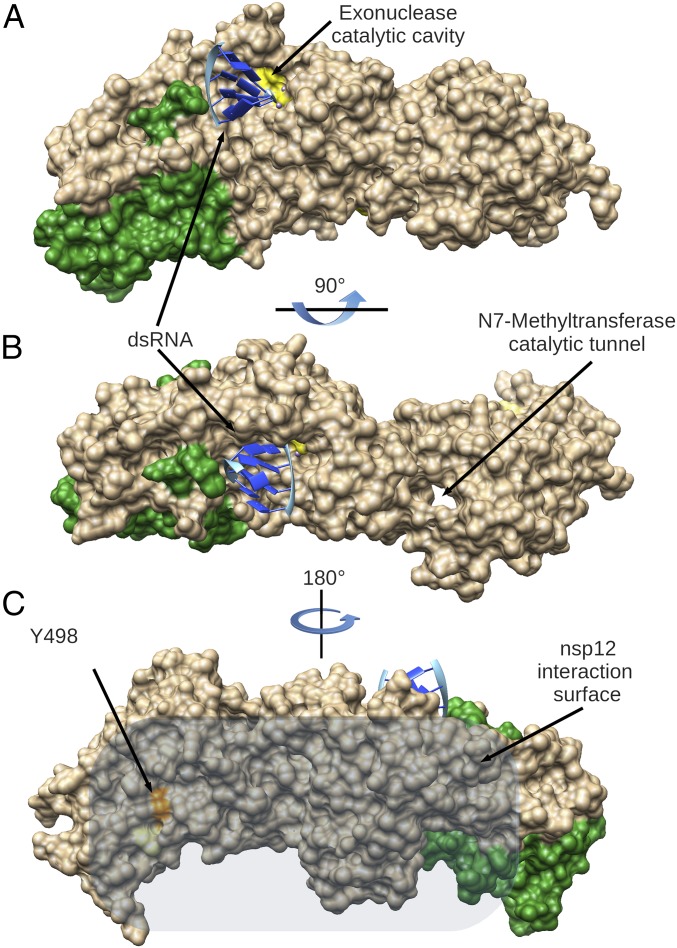
Model of the CoV RNA proofreading enzymes. Surface representation of the complex nsp10 (in green) with nsp14 (in beige), modeled with dsRNA (blue ribbon) in the exonuclease active site (yellow). (*A*) Side view of the complex showing the DEED ExoN catalytic site. (*B*) Top view of the complex showing the N7-MTase catalytic tunnel. (*C*) Proposed surface of interaction of nsp14 with the nsp12-RdRp. The position of the Y498 residue of nsp14 is highlighted in orange; when mutated in Ala, it abolishes the interaction with nsp12-RdRp.

Crystallographic and SAXS data show that nsp14, as a whole, is rather flexible and undergoes substantial conformational changes. These large movements may allow different functions and interactions with protein partners. The latter property is reminiscent of intrinsically disordered proteins, which have evolved binding platforms to perform sequential activities. Furthermore, the hinge may act as a molecular switch. Indeed, nsp14 is directly involved in at least two processes that use two different kinds of RNA substrates. During replication, newly synthesized dsRNA with a mismatch should be translocated from the RNA polymerase to the ExoN catalytic cavity of nsp14. During transcription, a nascent 5′-cap mRNA should go into the N7-MTase catalytic tunnel of nsp14 (Fig. 7). It was shown previously that the ExoN domain is closely involved in the activity of the N7-MTase (40). In the present study, we establish the reciprocity with the nsp14 N7-MTase domain that is essential for the ExoN activity. These results could be explained by alteration of the nsp14 flexibility. In fact, two conserved residues (Y296 and P297) located in the hinge of nsp14 and involved in the flexibility are essential for the ExoN activity. Altogether, these structural data combining structure and flexibility strengthen the unique feature of CoV nsp14.

Our results unveiled a second unexpected finding: The SARS-CoV polymerase complex exhibits a significantly lower fidelity compared with that of DENV RdRp, a virus with a genome one-third of its size. From this observation, we hypothesize that (*i*) the acquisition of nsp14-ExoN as an active part of the replication complex to increase fidelity has allowed relaxation of nucleotide selection by the viral RdRp, and (*ii*) as a consequence of this compensated low fidelity, larger RNA “coronavirus-like” genomes but encoding for a RdRp with much better intrinsic fidelity may exist; together with an associated ExoN, there might be further evolutionary space to satisfy Drake’s rule of approximately one mutation per replicated “larger” genome. Clearly, the origin and fate of nidovirus nsp14 sequences, structures, and activities may be significantly informative in the history of RNA-to-DNA genome evolutions.

In summary, we have reconstituted a functional pathway demonstrating that misincorporated nucleotides can be excised from the nascent viral genome. The conservation of nsp10, nsp12, and nsp14 across the CoV genus suggests that our finding may well apply to many members of the Coronaviridae family. Hence, a direct consequence of the CoV RNA proofreading system is its potential impact on antiviral strategies using nucleoside analogs, as illustrated by the excision of Rbv. Interestingly, the nsp10/nsp14-ExoN activity is inactive on dsRNA substrates bearing 3′-end ribose modifications (30). These results make the design of nonexcisable chain terminators a rational strategy for anti-CoV. Accordingly, Gilead’s GS-5734 1′-cyano–substituted nucleoside ribose analog was reported active against, at least, the MERS-CoV with an EC_50_ value of 0.34 μM (50). Very recently, the GS-5734 spectrum of action was extended to SARS-CoV, bat CoVs, and circulating human CoVs, making this nucleotide prodrug an effective pan-CoV inhibitor candidate (51). It will be interesting to assess if the ribose 1′-cyanyl group is also a determinant of resistance to ExoN activity.

Simultaneous inhibition of CoV RdRp and ExoN activities might also be a therapeutic option. It could restore antiviral potency of nucleotide analogs such as Rbv. Since viral nuclease enzymes (e.g., integrase from HIV, PA subunit from influenza virus) are compelling targets for antiviral therapy, designing nucleoside analogs and specific nuclease inhibitors concomitantly might prove a valid option against viruses endowed with RNA repair systems.

## Materials and Methods

### Expression and Purification of SARS-CoV Proteins.

All plasmids used in this study are described by Subissi et al. (16), except that SARS-CoV nsp12-RdRp was tagged with eight histidines. Bacterial protein expressions and purifications were done as previously described (16). The SARS-CoV polymerase complex (composed of nsp12/nsp8/nsp7) purification protocol included an immobilized metal affinity chromatography purification step in which the buffer was supplemented with 10 mM imidazole, and Co^2+^-bound proteins were eluted with 200 mM imidazole. A second chromatography step was performed on a Hiload 16/60 Superdex 200 column (GE Healthcare).

The SARS-CoV nsp14-coding sequence was cloned into expression vector pDEST14 (Thermo Fisher Scientific) to produce a recombinant protein carrying an N terminus His_6_-tag (18). Site-directed mutagenesis to generate plasmids expressing nsp14 mutants was performed using the QuikChange Site-Directed Mutagenesis Kit (Stratagene) according to the manufacturer’s instructions. Expression in bacteria and purification of nsp14 wt and mutants are described by Bouvet et al. (18).

### Crystallization and Structure Determination.

Crystallization was performed by the sitting-drop vapor diffusion method at 4 °C. Crystallization drops consisted of various ratios [1:1, 1:2, 2:1, and 2:2 (μL/μL)] of protein complex nsp10/nsp14 (4 mg/mL) in gel filtration buffer versus 600 μL of reservoir solution containing 0.1 M trisodium citrate (pH 5.5), 8% PEG 8000, and 30% hexanediol. Crystals were cryoprotected with a reservoir solution supplemented with 20% PEG 200, prior to being flash-cooled in liquid nitrogen. Diffraction data were collected at beamline Proxima1 at the Soleil synchrotron. The MAD data were collected to 3.5 Å resolution at the peak (λ = 1.28242 Å) and inflection (λ = 1.28282 Å) wavelengths for zinc. Higher resolution (3.38 Å) data were collected from a single crystal of the native complex (λ = 1.28348 Å). Datasets were processed and analyzed with the autoPROC toolbox (52), and the structure was solved by the MAD method using the anomalous scattering signal of zinc, with autoSHARP (53). Twenty zinc positions from the anomalous difference map helped calculate an initial electron density map and align the sequence identifying the key residues of zinc-binding sites. Auto-building with Buccaneer (54) was useful to extend the model, which was further manually built with Coot (55) and refined with BUSTER (56). The model was further refined with ROSETTA-PHENIX (57) to 3.38 Å, using the higher resolution native dataset. The final model has *R*_work_ = 19.6% and *R*_free_ = 23.4% and was confirmed to have good stereochemistry according to MOLPROBITY (58). Data collection and refinement statistics are listed in Table S1. All structure analysis and figures were done with UCSF Chimera (59).

### RNA Templates.

Synthetic RNAs (LS1, LS2, LS3, LS8, LS15, LS16, and LS17) were purchased from Biomers (HPLC grade), and LS2-Rbv was synthesized by Dharmacon/Thermo Fisher Scientific (HPLC grade). Table S3 provides sequences of these RNAs. RNA LS2, LS2-Rbv, and LS3 were radiolabeled with the T4 polynucleotide kinase (New England Biolabs) and [γ-^32^P]ATP (PerkinElmer), and are hereafter termed LS2*, LS2-Rbv*, and LS3*. LS2* was then annealed to either LS1, LS15, LS16, or LS17; LS2-Rbv* was annealed to LS15; and LS3* was annealed to LS8 by heating at 70 °C for 10 min and then cooling down to room temperature (with a primer/template ratio of 1.2:1).

### Polymerase Assays.

All polymerase reactions were preincubated for 30 min at 30 °C, containing 0.5 μM radiolabeled primer annealed to a template in reaction buffer [20 mM Tris (pH 8), 10 mM KCl, 1 mM DTT, 2 mM MgCl_2_] supplemented with the SARS-CoV polymerase complex [∼0.5 μM, as determined using UV absorption (λ = 280 nm) and Bradford analysis using BSA as a standard]. The percentage of active enzyme was estimated at <10% using titration of primer/template with increasing enzyme concentrations and manual burst analysis. Reactions were performed at 30 °C using indicated concentrations of either one NTP, Rbv-TP (Jena Bioscience), or all NTPs (at 500 μM each). For incorrect nucleotides, product formation followed a linear increase of incorporation product up to 20 min, and 18 min was chosen as a suitable time for steady-state kinetic studies. Reactions were quenched by the addition of an equal volume of loading buffer (formamide with 10 mM EDTA). RNA polymerization products were analyzed in polyacrylamide/7 M urea gels. RNA products were visualized using photostimulated plates and a phosphorimager (Fuji).

### Sequencing of cDNA Clones from Newly Polymerized RNA Products.

RNA products to be sequenced were excised and electroeluted using a GEBAFLEX extraction kit (Interchim), following the manufacturer’s instructions. Recovered RNAs were precipitated and resuspended in H_2_O, and RT was performed with a forward primer (5′-GTATCCCCATCTCATTTTA-3′) using a OneStep RT-PCR Kit (QIAGEN). After heating to 95 °C (to switch from the RT reaction to the PCR, following the manufacturer’s instructions), the reverse primer was added (5′-GTCATTCTCCTAAGAAGC-3′). Cloning of the PCR products was performed with the CloneJET PCR Cloning Kit (Thermo Fisher Scientific), and plasmid DNAs were sequenced using a third primer (5′-CGATGAGTTTTCGGTATTATC-3′) by Eurofins Genomics. The reported *P* value was calculated using a Z-test for comparison of two proportions.

## Supplementary Material

Supplementary File
