# Supplementary material for: Structural and molecular basis of mismatch correction and ribavirin excision from coronavirus RNA
Source: Proc Natl Acad Sci U S A. 2017 Dec 26;115(2):E162–71. doi: 10.1073/pnas.1718806115 (PMC5777078; doi:10.1073/pnas.1718806115)
Supplement: Supplementary File [file pnas.201718806SI.pdf]

Supporting Information

Ferron et al. 10.1073/pnas.1718806115

SI Text

SAXS Experiments and Data Processing. All SAXS measurements were carried out at the European Synchrotron Radiation Facility on beamline BM29 at a working energy of 12.5 keV. Data were recorded using the Pilatus 1M pixel detector at a sample-detector distance of 2.867 m, leading to scattering vector q, ranging from 0.025 to 5 nm^{-1}. The scattering vector is defined as $q = 4\pi/\lambda \sin\theta$, where λ is the wavelength of the incident radiation in nanometers and θ is half of the angle between the incident and scattered radiation. A range of six concentrations of nsp14 was measured and analyzed (0.53, 0.89, 1.39, 2.09, 3.47, and 5.24 mg/mL). The protein buffer composition was 30 mM Hepes (pH 7.5), 300 mM NaCl, 5% glycerol, and 1.5 mM DTT. All samples were centrifuged for 15 min at 15,000 × *g*, before measurement to minimize contributions from aggregated particles. A 40-μL protein sample was injected in a 1.8-mm capillary with flow to minimize radiation damage, and data were collected at 283 K. Ten frames of 1 s each were made for each protein concentration and were combined to give the average scattering curve for each measurement. As a control, the buffer data were collected for reference before and after measurement for each protein sample, in the same conditions. The forward scattering intensity was calibrated using BSA as a reference at 5 mg/mL.

Data were processed with the ATSAS package (1) according to the standard procedure. Ten 1-s frames were averaged by protein using the PRIMUS program (2), any frames affected by radiation damage were excluded, and the buffer background runs were then subtracted from the sample runs. Data from each concentration were scaled and merged to create a chimeric curve using all data. The values of total forward scattering at zero angle, $I_{(0)}$, and the radius of gyration, R_g, were calculated from the experimental SAXS profiles using Guinier approximation, which is valid in the range of $R_g < 1.3$. These parameters and the maximum particle dimension, D_{max}, were also computed from the distribution distance function, $p(r)$, using GNOM (3). The molecular mass (MM) was calculated from the SAXS data using the value of $I_{(0)}$ combined with protein concentration relative to a BSA standard, as well as from the concentration-independent excluded Porod volume. The latter was determined given that the empirical ratio between the Porod volume and MM of a protein is ~1.7 (4). The low-resolution shape of nsp14 was reconstructed ab initio using the program DAMMIF (5). Forty independent bead models were built. These models were compared and superimposed using the DAMAVER suite. Align models were averaged and finally filtered to obtain a model with a volume comparable to analyzed data (6). The theoretical scattering intensity of nsp14 from nsp14/nsp10 high-resolution structure was calculated using CRYSOL software to compare nsp14 crystallographic structure with an nsp14 low-resolution SAXS model (7). The SREFLEX program was used to estimate the flexibility of nsp14 crystallographic structure and to improve its agreement with experimental SAXS data (8).

1. Petoukhov MV, Svergun DI (2007) Analysis of X-ray and neutron scattering from biomacromolecular solutions. *Curr Opin Struct Biol* 17:562–571.
2. Konarev PV, Volkov VV, Sokolova AV, Koch MHJ, Svergun DI (2003) *PRIMUS*: A Windows PC-based system for small-angle scattering data analysis. *J Appl Crystallogr* 36:1277–1282.
3. Svergun DI (1992) Determination of the regularization parameter in indirect-transform methods using perceptual criteria. *J Appl Crystallogr* 25:495–503.
4. Petoukhov MV, et al. (2012) New developments in the ATSAS program package for small-angle scattering data analysis. *J Appl Crystallogr* 45:342–350.
5. Franke D, Svergun DI (2009) DAMMIF, a program for rapid ab-initio shape determination in small-angle scattering. *J Appl Crystallogr* 42:342–346.
6. Volkov VV, Svergun DI (2003) Uniqueness of *ab initio* shape determination in small-angle scattering. *J Appl Crystallogr* 36:860–864.
7. Svergun D, Barberato C, Koch MHJ (1995) CRYSOL: A program to evaluate X-ray solution scattering of biological macromolecules from atomic coordinates. *J Appl Crystallogr* 28:768–773.
8. Panjkovich A, Svergun DI (2016) Deciphering conformational transitions of proteins by small angle X-ray scattering and normal mode analysis. *Phys Chem Chem Phys* 18: 5707–5719.
9. Imbert I, et al. (2008) The SARS-coronavirus PLnc domain of nsp3 as a replication/transcription scaffolding protein. *Virus Res* 133:136–148.
10. Bouvet M, et al. (2012) RNA 3′-end mismatch excision by the severe acute respiratory syndrome coronavirus nonstructural protein nsp10/nsp14 exoribonuclease complex. *Proc Natl Acad Sci USA* 109:9372–9377.
11. Bouvet M, et al. (2010) In vitro reconstitution of SARS-coronavirus mRNA cap methylation. *PLoS Pathog* 6:e1000863.

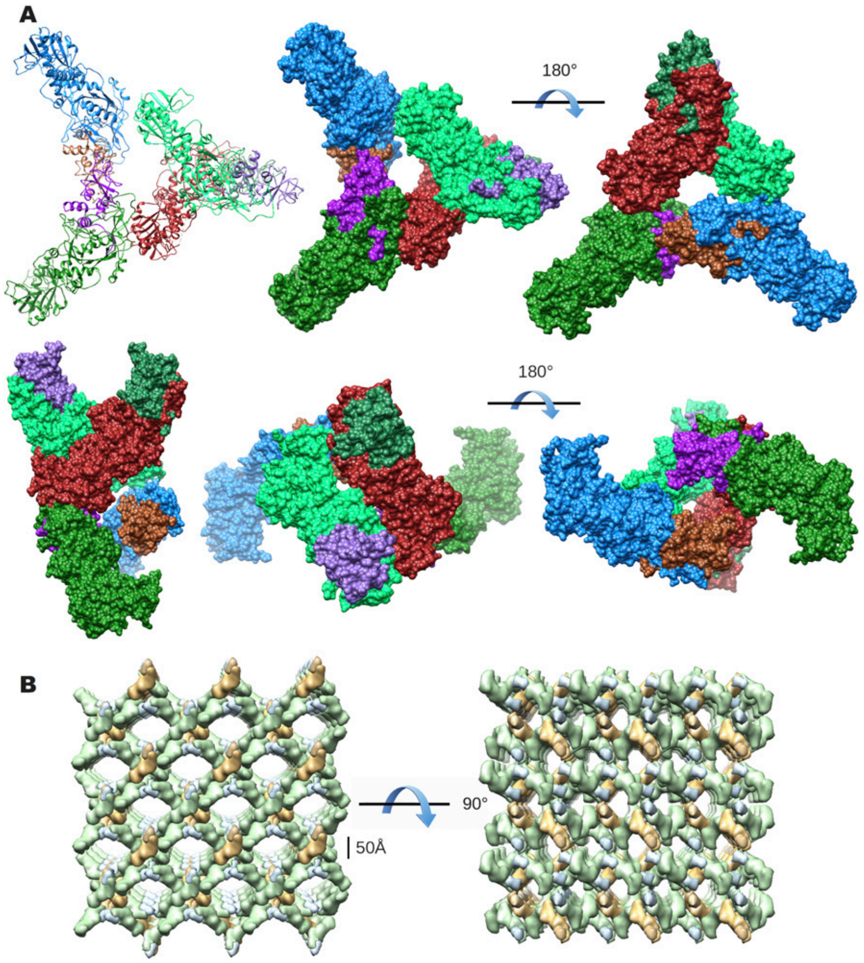

Fig. S1. (*A*) Asymmetrical unit formed of four-heterodimer nsp10/nsp14 in different orientations. Colors for nsp10 are dark green (chain M), purple (chain N), brown (chain O), and light purple (chain P); colors for nsp14 are firebrick (chain A), green (chain B), light green (chain C), and blue (chain D). (*Top Left*) Ribbon assembly and several views of surface counterparts in different orientations are shown. (*B*) Analysis of the structure of the crystal allows us to observe large oval-shaped solvent canals of ~120 by ~60 Å running through. These large canals are probably the reason for the low crystal diffraction. Nsp14 molecules are colored green, and nsp10 molecules are colored yellow.

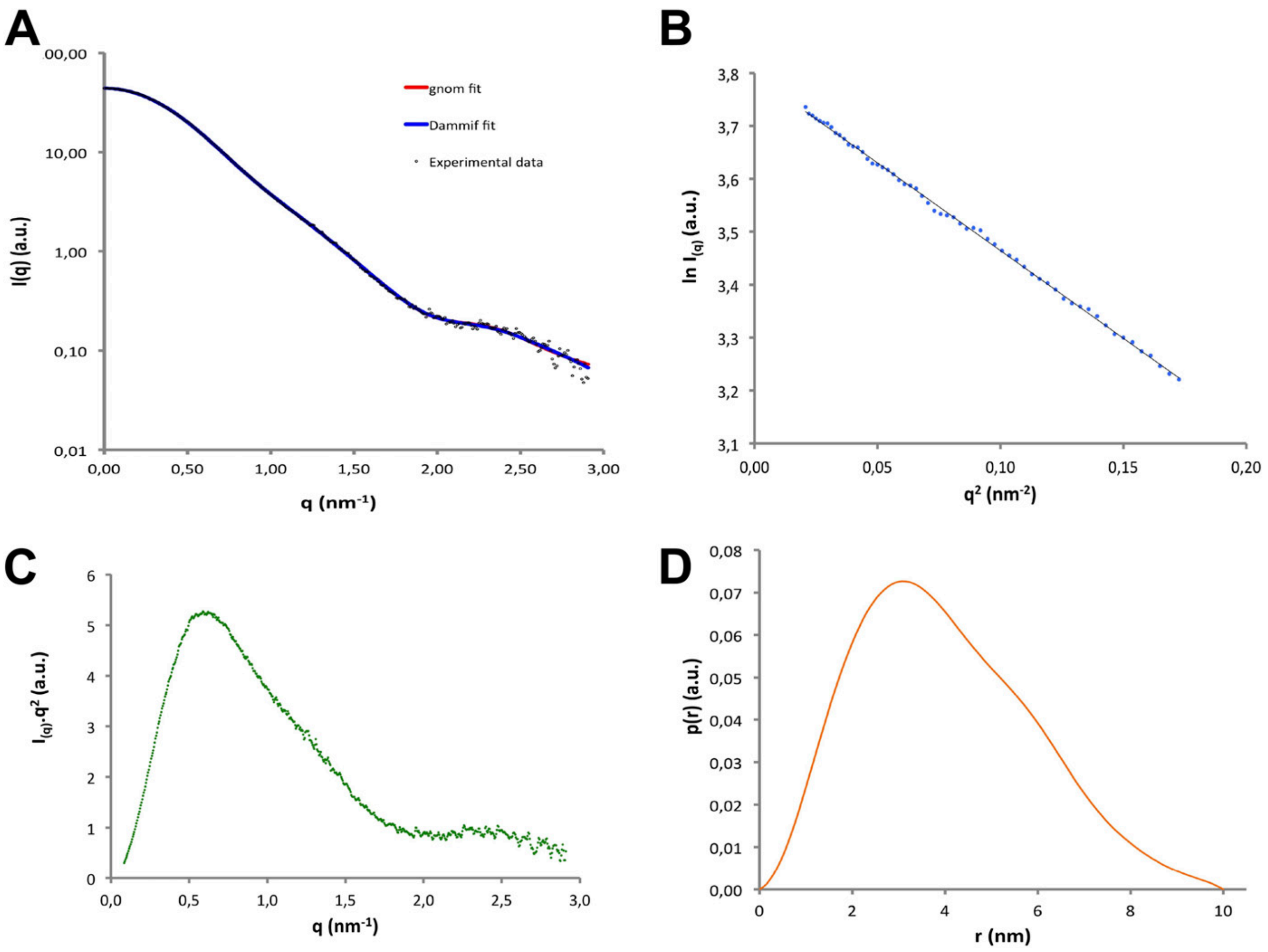

Fig. S2. SAXS analysis of SARS-CoV nsp14. (*A*) Nsp14 X-ray scattering curves and fitting. Experimental data are represented as black dots; the GNOM theoretical fit (red) and DAMMIF fitting (blue) of the most probable model among 40 tested samples are shown. (*B*) Guinier plot (blue) and linear regression (black) are shown. No aggregation is detected. (*C*) Kratky plot based on the SAXS data. Curves present a maximum at small angles and resume to 0 toward large angles, indicating a globular protein. (*D*) Pair distribution function of nsp14. D_{max} = 10 nm.

A

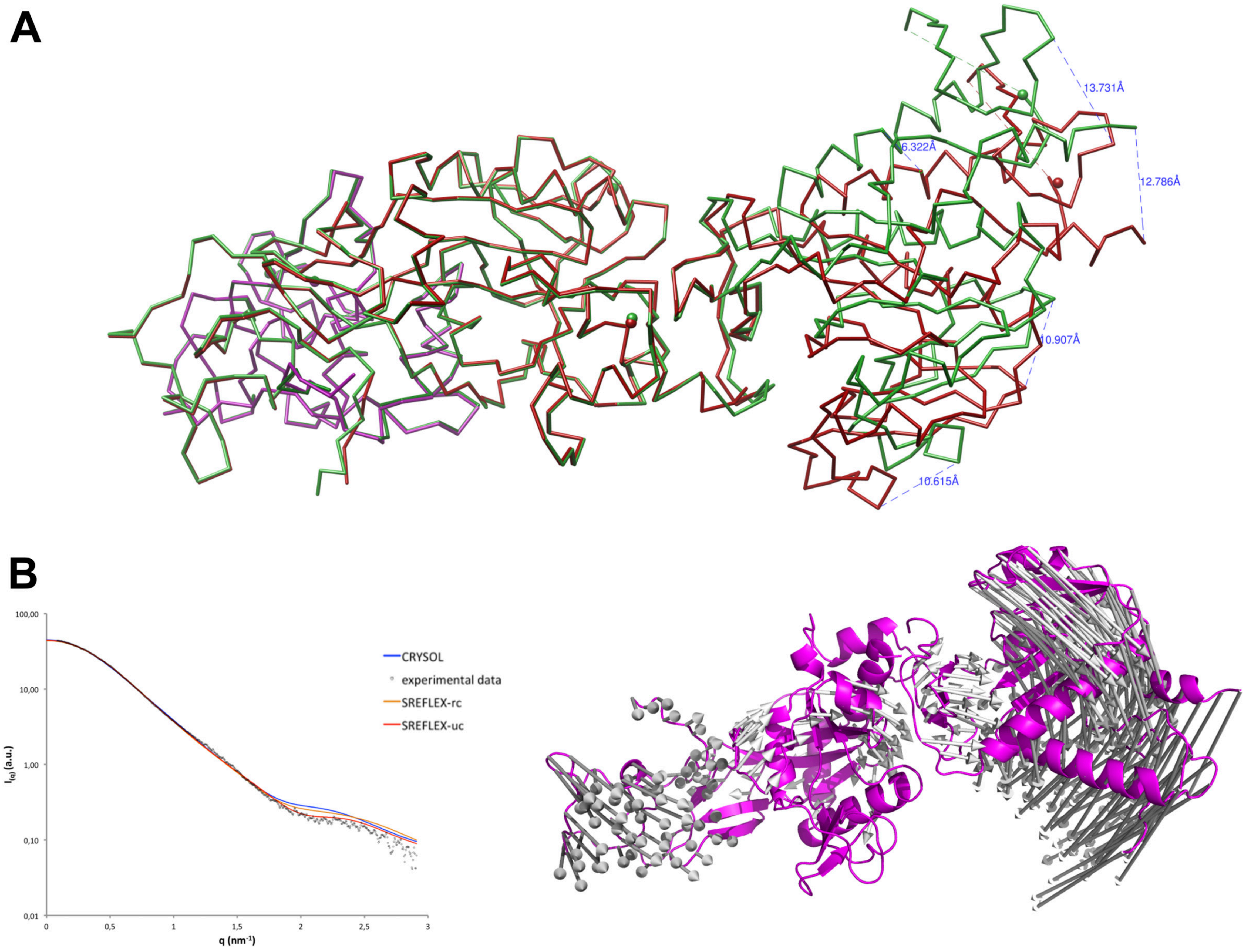

Fig. S3. (*A*) Superimposition of two nsp10/nsp14 chains present in the asymmetrical unit. If the nsp14 N-terminal domain, together with nsp10, superimposed well, the nsp14 C-terminal domain presents an ~10 to ~14 Å deviation. (*B*) Comparison between the experimental SAXS data, the nsp14 X-ray structure theoretical profile, and the SREFLEX-simulated nsp14 profile with (rc) or without (uc) secondary structure restraints. The theoretical profile of nsp14 in complex with nsp10 does not fit with the experimental data of nsp14 alone in solution. To estimate and probe large-scale motions in nsp14, we used normal mode analysis on the best peptide chain of nsp14. We improved profiles in better agreement with experimental data. Unrestrained simulations (red curve) for which all of the subdomains are assumed flexible, show major conformational changes both in the N terminus and C terminus. The ribbon structure of nsp14 represented with vectored SREFLEX-simulated movements was obtained after completion of both refinement stages (restrained and unrestrained). The vectors show that, in solution, the nsp14 N-terminal domain (nsp10-binding site) and the entire C-terminal domain are prone to ~10 to ~14 Å deviation.

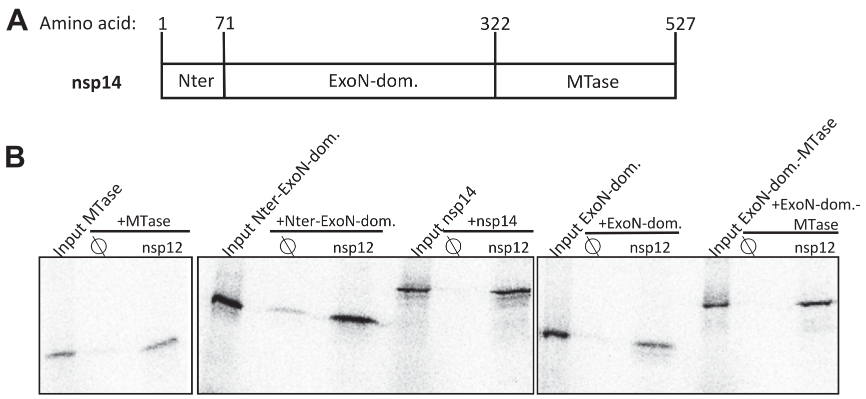

Fig. S4. GST pull-down analysis to study protein interaction between nsp12-RdRp and nsp14 domains. (*A*) Nsp14 domain boundaries used in this study are shown. (*B*) Each panel shows an autoradiogram from an SDS/PAGE analysis of in vitro-translated nsp14 domains pulled down by binding to either empty glutathione-Sepharose beads (lanes ∅) or GST-nsp12 (lanes nsp12), as previously described (9). Equal amounts of [^{35}S]methionine-labeled target were used for lanes (∅ and nsp12), and the input material was also run next to the pull-down samples, as indicated for each panel. Each interaction was independently tested three times. dom., domain; Nter, N terminus.

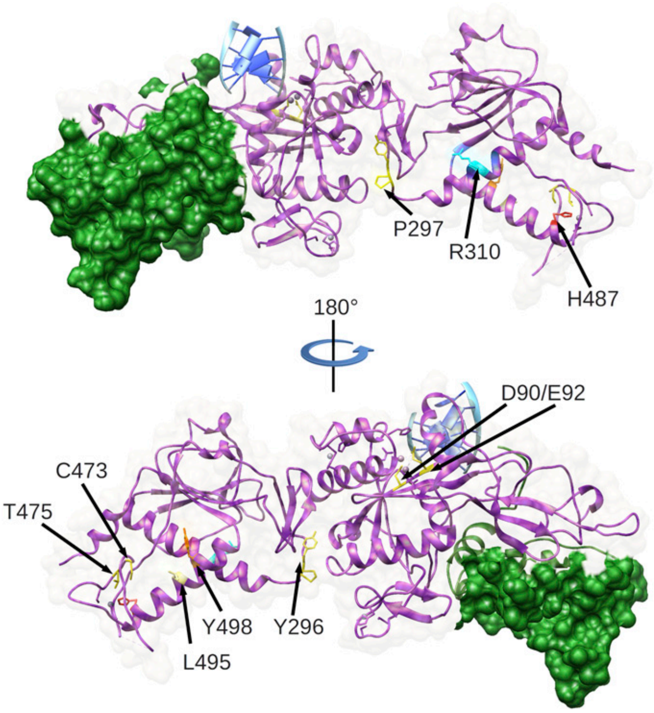

Fig. S5. Mix representation of the nsp10 (green surface)/nsp14 (purple ribbon) complex, modeled with dsRNA (blue ribbon) in the exonuclease active site. Highlighted are R310, a residue critical for SAM binding, and D90/E92 catalytic residues of the ExoN activity. Other residues are those that have been shown to be essential for ExoN activity.

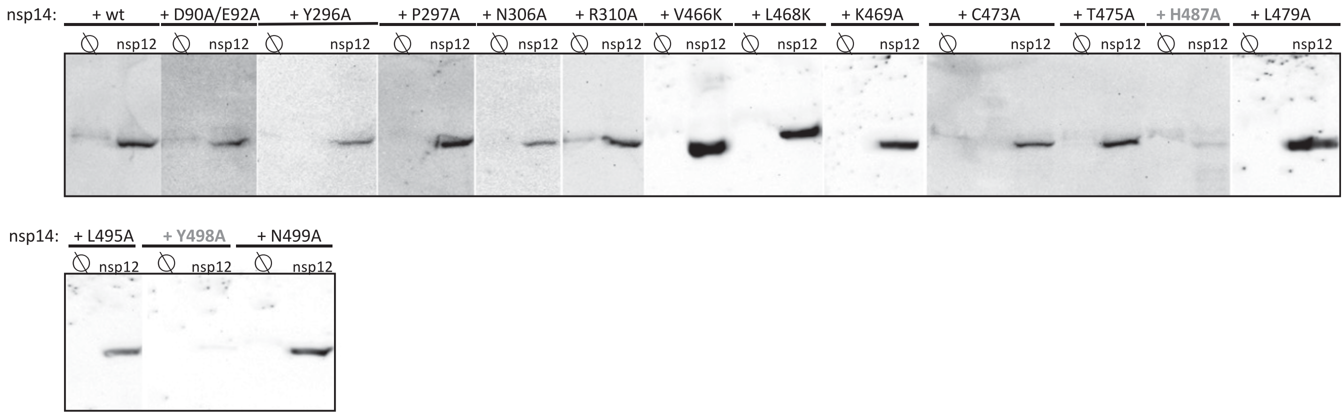

Fig. S6. GST pull-down analysis to study protein interaction between nsp12-RdRp and nsp14 mutants. Immunoblots of nsp14 wt or mutant pulled down by binding to either empty glutathione-Sepharose beads (lanes ∅) or GST-nsp12 (lanes nsp12) are shown, as previously described (9). Briefly, GST-nsp12 was bound to glutathione-Sepharose beads and incubated with 10 μg of SARS-CoV nsp14 wt or mutants, all carrying an amino-His_6-tag. As a negative control, empty beads were also incubated with the different mutant proteins. Pull-down experiments were next revealed by Western blot with anti–penta-His antibody. GST pull-down experiments have been done three independent times.

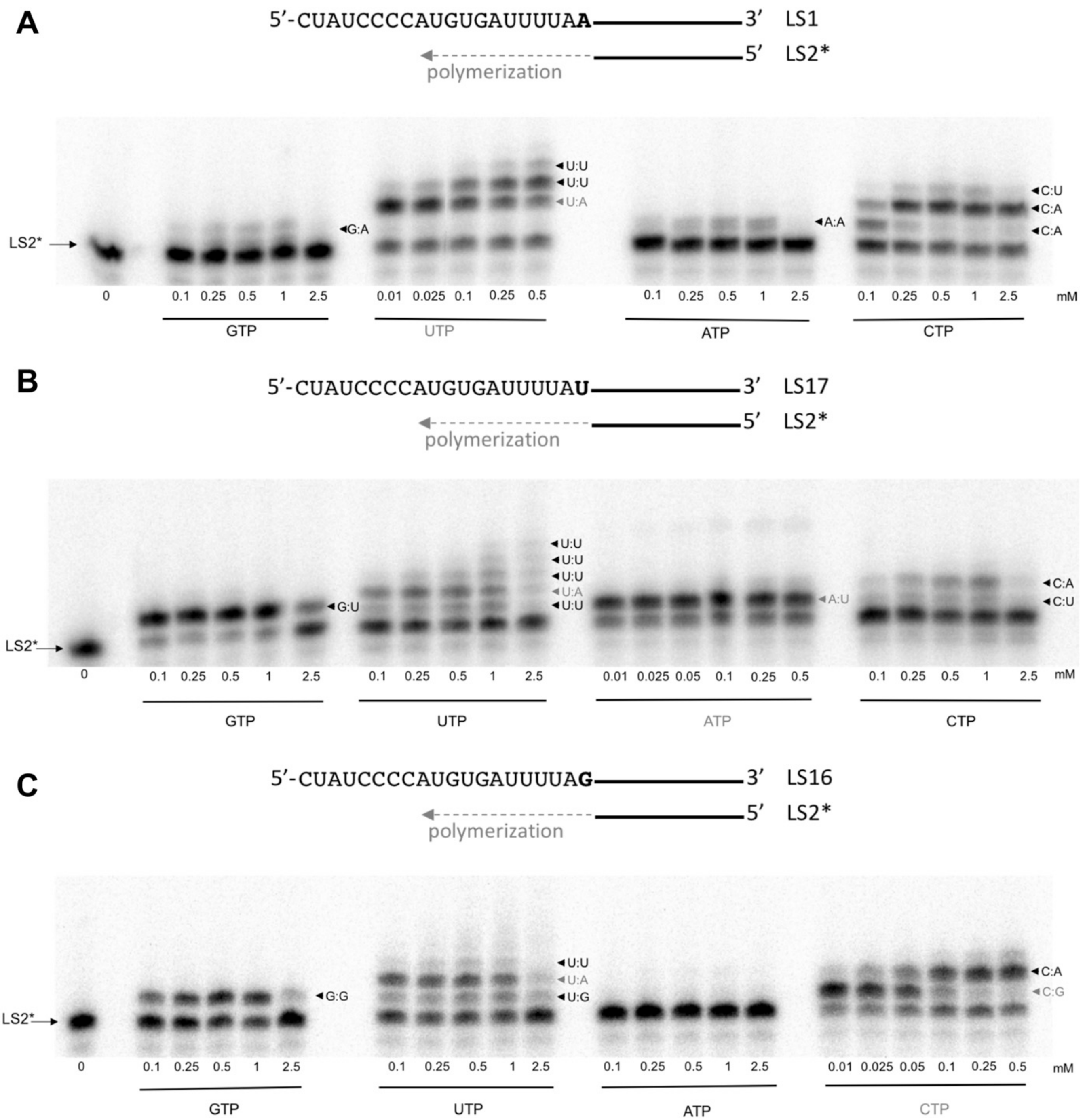

Fig. S7. Single-nucleotide incorporation catalyzed by the SARS-CoV polymerase complex using different RNA primers/templates. LS2*/LS1 (*A*), LS2*/LS17 (*B*), and LS2*/LS16 (*C*) RNA substrates were used with increasing concentrations of the indicated NTP. Single-nucleotide primer extensions were performed in the same experimental condition as in Fig. 4. Watson–Crick base pairs are indicated in gray, and mismatches are indicated in black.

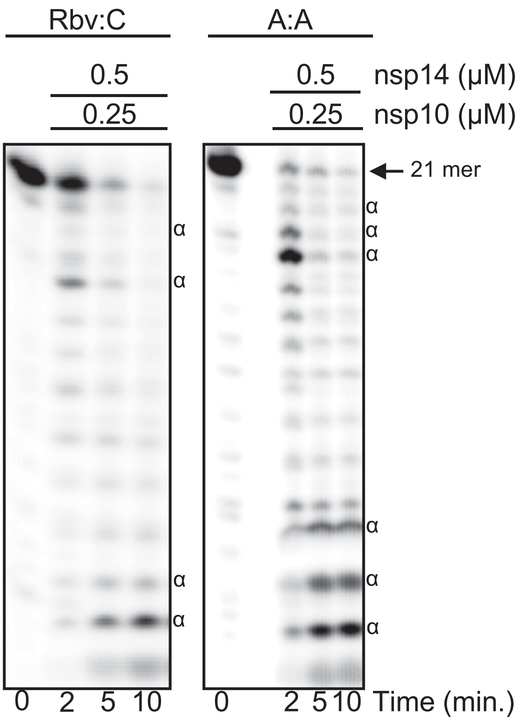

Fig. S8. Nsp10/nsp14-ExoN excision of Rbv:C and A:A base pairs in RNA substrates. Time course of 0.5 μM of primer*/template excision: LS2-Rbv*/LS15 bearing an Rbv:C and LS3*/LS8 bearing an A:A mismatch, in the presence of nsp10 and nsp14 (concentrations indicated in the panel). Nuclease reactions were run as described previously (10). α, dsRNA degradation products by the nsp14-ExoN activity.

Table S1. Data collection and refinement statistics

Datasets	Native	Zn phased crystal	
Data collection			
Space group	$P2_12_12$	$P2_12_12$	
Cell dimensions			
a, *b*, *c*; Å	185.88, 189.79, 196.24	186.01, 189.53, 195.57	
α, β, γ; °	90.0, 90.0, 90.0	90.0, 90.0, 90.0	
		Peak	Inflection
Wavelength, Å	1.28348	1.28242	1.28282
Resolution, Å	94.89–3.38 (3.39–3.38)	63.53–3.57 (3.58–3.57)	63.53–3.87 (3.88–3.87)
R_{merge}	0.129 (1.013)	0.164 (1.17)	0.034 (0.98)
I/σ	15.59 (2.27)	13.2 (2.0)	12.3 (2.3)
Completeness, %	99.6 (99.97)	98.2	97.4
Multiplicity	9.0 (9.3)	3.9	3.7
CC(1/2)	0.997 (0.77)		
Refinement			
Resolution, Å	64.04–3.38 (3.50–3.38)		
No. reflections	96,922		
R_{work}/R_{free}	0.19/0.23		
No. atoms			
Protein	20,340		
Ligand/ion	41		
Water	247		
B-factors			
Protein	97.50		
Ligand/ion	119.48		
Water	88.41		
Rmsds			
Bond lengths, Å	0.016		
Bond angles, °	1.98		

Values in parentheses are for the highest resolution shell.

Table S2. Effects of a variety of nsp14 mutations on the nsp14/nsp12-RdRp interaction, and on ExoN and N7-MTase enzyme activities

Nsp14 mutants	Interaction with nsp12-RdRp*	Activity ExoN,† %	Activity N7-MTase,‡ %
Wt	+	100	100
D90A/E92A	+	**0.25 ± 0.07**	106 ± 3.3
Y 296 A	+	**1.7 ± 0.5**	64 ± 1.3
P 297 A	+	**12.4 ± 3.1**	102 ± 0.7
N 306 A	+	61.7 ± 7.3	111 ± 2.4
R 310 A	+	79 ± 55.4	**1 ± 0.1**
V 466 K	+	140.1 ± 31.7	108 ± 0.7
L 468 K	+	109.5 ± 38	110 ± 2.9
K 469 A	+	72.4 ± 34	109 ± 3.1
C 473 A	+	**5 ± 0.8**	92 ± 1.6
T 475 A	+	**8.6 ± 2.9**	106 ± 9.5
L 479 A	+	89.33 ± 2.5	107 ± 2.5
**H 487 A**	–	**0.7 ± 0.05**	**14 ± 2.5**
L 495 A	+	**2 ± 0.5**	**26 ± 0.7**
**Y 498 A**	–	**1.3 ± 0.04**	95 ± 6
N 499 A	+	85 ± 21.7	105 ± 5.4

*Interactions between nsp12 and nsp14 mutants were analyzed by GST pull-down, with primary data presented in Fig. S6. This method is not quantitative: The (+) symbol refers to an interaction detected after incubation with GST-nsp12 but not with empty beads, and the (–) symbol refers to an interaction detected with neither GST-nsp12 nor empty beads. The two nsp14 residues important for the interaction with nsp12 are highlighted in gray.

†For ExoN activity quantification, equal amounts of nsp14 mutants (100 nM) were incubated with nsp10 (600 nM) and *p-H4 RNA in the buffer reaction described by Bouvet et al. (10). After 2 min and 20 min of incubation, respectively, reaction products were separated using urea-PAGE, revealed using a FujiImager, and quantified with Image Gauge software. Results are presented as the percentage of ExoN activity (nsp10/nsp14 wt was taken as 100% of activity).

‡N7-MTase activity of nsp14 wt or mutants was measured as described previously (11). The N7-MTase activity of nsp14 wt was set up at 100%.

Enzymatic activity measurements were performed in triplicate. Loss of enzymatic activity is shown in bold.

Table S3. RNA sequences of synthetic RNAs used in this study

RNA name	5′-3′ sequence
LS1	CUAUCCCCAUGUGAUUUUAAUAGCUUCUUAGGAGAAUGAC
LS2	GUCAUUCUCCUAAGAAGCUA
LS2-Rbv	GUCAUUCUCCUAAGAAGCUARBV
LS3	GUCAUUCUCCUAAGAAGCUAA
LS8	UAUCCCCAUCUCAUUUUAAUAGCUUCUUAGGAGAAUGAC
LS15	CUAUCCCCAUGUGAUUUUACUAGCUUCUUAGGAGAAUGAC
LS16	CUAUCCCCAUGUGAUUUUAGUAGCUUCUUAGGAGAAUGAC
LS17	CUAUCCCCAUGUGAUUUUAUUAGCUUCUUAGGAGAAUGAC

Rbv, ribavirin monophosphate.
